# LOXL2 labels inflammation-associated myofibroblasts predicting kidney allograft dysfunction and fibrosis

**DOI:** 10.3389/fimmu.2025.1671117

**Published:** 2026-01-13

**Authors:** Paula Schütz, Birte Hüchtmann, Veerle Van Marck, Barbara Heitplatz, Carolin Walter, Rebecca Rixen, Hermann Pavenstädt, Stefan Reuter, Konrad Buscher

**Affiliations:** 1 Department of Medicine D, Division of General Internal Medicine, Nephrology and Rheumatology, University Hospital of Münster, Münster, Germany; 2 Institute of Pathology, University of Münster, Münster, Germany; 3 Institute of Medical Informatics, University of Münster, Münster, Germany

**Keywords:** kidney, transplantation, allograft, fibrosis, myofibroblast

## Abstract

Progressive allograft fibrosis remains a major obstacle in kidney transplantation. Early identification of patients at high risk could be instrumental to improve outcomes. Here, we investigated Lysyl oxidase like 2 (LOXL2) as a biomarker for graft fibrosis and dysfunction. Using single-cell sequencing and imaging of transplant biopsies, we found that LOXL2 labeled an intertubular myofibroblast-like cell type with a smooth muscle actin (SMA)-negative, CD68-positive phenotype and high extracellular matrix activity. These cells were present in non-fibrotic and fibrotic regions using collagen 3 as a scaffold. Native kidneys also harbored LOXL2+ myofibroblasts, albeit at much lower levels. Following transplant surgery, LOXL2+ cells could rapidly emerge within days, particularly during episodes of rejection, where they associated with leukocyte aggregates. Elevated cell numbers were not irreversible as shown in follow-up biopsies. A retrospective analysis of 118 biopsies revealed a significant association with fibrosis, inflammation, and kidney function but not with other Banff parameters. Non-rejecting allografts displayed high variability in LOXL2+ cells, with high abundance serving as a long-term predictor of reduced allograft function. Our findings point to a new subset of inflammation-associated myofibroblasts that may be useful as a biomarker for early fibrogenesis.

## 1 Introduction

Progressive interstitial fibrosis represents a significant challenge in kidney transplantation (1, 2). It frequently results in a progressive decline in graft function, proteinuria, hypertension, and ultimately, the loss of the transplant (2).

The pathogenesis of graft fibrosis is complex, involving both immune-mediated and non-immune-mediated factors (3, 4). Sequential protocol biopsies have demonstrated that it can develop rapidly, with mean fibrosis levels reaching 27% after 3 months and 32% after 12 months post-transplantation (5, 6). These findings underscore the critical role of immune activity in the early stages of fibrosis and highlight the need for timely intervention to prevent long-term graft deterioration. It includes reducing ischemia-reperfusion injury, minimizing cardiovascular risk factors, controlling calcineurin-inhibitor exposure, screening for recurrence of the primary renal disease, and reducing episodes of graft rejection.

Graft dysfunction ultimately results from glomerular sclerosis, tubular atrophy, and interstitial fibrosis—common end-stage patterns of injury. Interstitial fibrosis is characterized by extensive, non-reversible extracellular matrix (ECM) deposition and tissue scarring driven by activated fibroblasts and myofibroblasts. A recent study has highlighted the potential of urinary renal progenitor cells as correlates of defective regenerative mechanisms, which can predict graft dysfunction, mesangial expansion, and fibrosis (7). However, reliable biomarkers for patients at high risk are still largely lacking.

We previously utilized unsupervised transcriptome-driven clustering to identify seven distinct immune phenotypes in human kidney allografts (8). Two of these phenotypes were strongly associated with fibrosis and poor graft survival. Building on this framework, we identified lysl oxidase like 2 (LOXL2) within a fibroblast-/macrophage-associated gene signature. It catalyzes matrix remodeling by cross linking collagen fibers, and has been implicated in fibrotic diseases and cancer metastasis (9). In the present study, our objective was to investigate the cellular origin of LOXL2 in kidney transplants, its role in graft biology, and its clinical utility as a biomarker for progressive fibrosis.

## 2 Materials and methods

### 2.1 Antibodies

All antibodies are listed in Supplementary data 1.

### 2.2 Patient selection

We included patients hospitalized for kidney transplant injury at the University Hospital Münster (Germany). All patients consented to the study which was approved by the state ethics committee (Ethik-Kommission Ärztekammer Westfalen-Lippe and Universität Münster, 2011-400-f-S, 2019-337-f-S). The indication for a graft biopsy was unexplained graft injury as assessed by creatinine or proteinuria. The standard immunosuppressive regimen comprised tacrolimus, mycophenolate, and prednisolone. Exclusion criteria were coinfections. Patient demographics were obtained from the hospital database. We screened two cohorts of kidney transplanted patients with indication biopsies for inclusion in this study. The first and second cohort included biopsies between February 2012 and March 2018 (n=53), and between January 2020 and November 2020 (n=84), respectively. 19 biopsies were excluded because of technical issues or exclusion criteria. This resulted in n=118 biopsies for analysis. Of these, n=103 had a transplant duration greater than 28 days and n=15 shorter or equal 28 days. Both data sets were analyzed for batch effects and outliers. A principal component analysis demonstrates a large overlap between both data sets but the 2012–2018 cohort is somewhat more restricted with less variance.

### 2.3 Allograft biopsies and specimen handling

Two 17G needle biopsies were obtained from each transplant following standard operating procedures. For Banff scoring, immunohistochemistry and immunofluorescence microscopy, the specimens were immediately stored in formaldehyde. After sectioning, hematoxylin and eosin and/or Periodic acid–Schiff staining were performed according to standard histology protocols in collaboration with the Institute of Pathology (University of Münster, Germany). In an explorative approach, we used 18 subsequent biopsies to perform single-cell sequencing of one of the two specimens. Here, the sample was directly stored in sodium chloride 0.9% at 4°C, and within 1 hour at -80°C in CryoStor CS10 (Sigma Aldrich) medium using a freezing container (-1 °C/min).

### 2.4 LOXL2 staining, counting and thresholding

Immunohistochemistry was performed on formalin-fixed, paraffin-embedded sections using the Ventana BenchMark Ultra platform (Roche) applying a primary anti-human LOXL2 antibody (rabbit anti-human, polyclonal, catalog number HPA036257; Sigma Aldrich/Merck, dilution 1:50; **Supplementary Table 1**). In the renal cortex from each biopsy all LOXL2-positive cells in the intertubular interstitium were counted using a 20x magnification covering a field of view (FOV) of 0.6 - 0.7 mm^2^. Several FOV were counted and averaged per biopsy (average LOXL2+ cells per reference FOV) with the following criteria: only cortex area, not adjacent to the capsule, only intertubular cells in the peri- and intertubular interstitium with dendritic cell shapes, counting of as many FOV as possible but at least three. Representative images were acquired using an upright Zeiss light microscope and the Diskus Imaging Software (Hilgers Technisches Büro, Germany).

Counting was performed by two independent nephropathologists blinded to the histological and clinical diagnosis. They independently counted LOXL2+ cells in 31 samples and the Intraclass Correlation Coefficient (ICC) and Bland–Altman analysis (10) was calculated to test for reproducibilty. Agreement between observers was good (ICC = 0.864; 0.75–0.90 is considered good and > 0.9 excellent). Bland–Altman analysis showed a mean bias of 0.33 with 95% limits of agreement from −13.31 to 13.97, indicating wider discrepancies at higher counts. A two-sided paired t-test found no systematic difference between the two observers (p = 0.793). Pearson correlation was high (r = 0.920, p < 0.001).

We defined a LOXL2+ cell threshold to clearly separate normal and pathologically altered kidneys. The maximum number in healthy, non-transplanted kidneys was 21 cells/FOV. In 103 tested allograft biopsies, the mean LOXL2+ cell count was 22. In addition, the ideal cutoff was also evaluated based on the ROC analysis and Youden’s Index in the 8 and 19 months prediction data set. Both calculations yielded the optimal cutoff of 27 LOXL2+ cells/FOV. These considerations led to the definition of a 30-cell threshold to define pathologically elevated LOXL2+ cell numbers.

A different antibody was also used (Abcam ab96233, polyclonal rabbit anti-human LOXL2; **Supplementary Table 1**) with a similar staining protocol to confirm the specificity of the intertubular cell stainings. Side by side comparison of both antibodies in two adjacent slices of one biopsy was performed by blinded nephropathologists, and showed largely similar results (R^2^ = 0.87, p = 0.002).

### 2.5 Statistics

The data were analyzed for normal distribution using the Shapiro-Wilk and Kolmogorov-Smirnov tests. The Kruskal-Wallis test was used to determine the correlation of the number of LOXL2+ cells and different nominal variables. To assess the correlation of the number of LOXL2+ cells with bivariate nominal variables Mann-Whitney-U Test was performed. Ordinal and metric variables were correlated with LOXL2+ cell numbers using Spearman-Rho test and Kendall-Tau, respectively.

### 2.6 Immunofluorescence microscopy

Deparaffinization was achieved by sequential washing with xylene, 100% ethanol, 96%, 80% and 70% for 5 minutes each. Antigen retrieval was performed with 10 mM citrate buffer containing Tween 20 in a microwave (700 W). Sections were then cooled down, incubated with 0.5 mg/ml pronase at 37 °C, and digestion was stopped with 2% glycine. Samples were washed once with PBS, blocked with 1% BSA, and incubated with primary antibodies overnight at 4 °C. The next day, samples were washed with PBS and incubated with fluorophore-conjugated secondary antibodies for 2 hours at room temperature. After incubation, the samples were washed and mounted using a DAPI-containing mounting medium. We co-stained LOXL2 with the anti-human antibodies PDGFRb, CXCL12, COL1A1, COL3A1, CD45, MHC-II, CD68 and smooth muscle actin (SMA), and used isoform antibodies as controls (**Supplementary Table 1**). Images were acquired with a Zeiss Axio Imager M2 microscope at low magnification (10×/20x) or a Zeiss spinning disc confocal microscope at high resolution (40×) using the Zeiss Zen software.

### 2.7 Prediction of allograft dysfunction

Samples with a graft age < 28 days were excluded. The remaining 103 biopsies were screened for follow up creatinine measurements in the hospital data base. We defined two time periods: 2–8 months after biopsy, and 11–19 months after biopsy. The baseline creatinine concentrations in these time periods were determined as average. Samples were excluded that did not have follow up data in these time periods, or showed high variability of the creatinine measurements within the time periods, excluding the definition of a baseline creatinine. Samples were categorized based on the creatinine increase of > 0.5 mg/dl compared to the baseline creatinine concentration before biopsy. This cutoff was chosen because a persistent increase of 0.5 mg/dl showed the highest hazard ratio for graft failure (11). Univariate and multivariate logistic regression analysis and receiver operator characteristic (ROC) analysis were calculated for non-rejection biopsies (**Tables 1**, **2**).

**Table 1:** Clinical and histological characterization of kidney allograft biopsies stratified by the number of LOXL2+ cells.

Mean LOXL2+ cells/FOV	< 30	>= 30
total biopsies, n	90	28
Patient characteristics		
male	*61 (68%)*	16 (57%)
female	*29 (32%)*	12 (43%)
Age (years)		
median (min-max)	*53 (16-81)*	48,5 (18-71)
Days post transplant		
median (min-max)	*740 (2-13217)*	757 (8-7255)
Serum creatinine, baseline		
median (min-max)	*1,6 (0,75-10)*	*2,3 (1,2-4)*
Baseline creatinine, 2-8 months after biopsy		
n	84	*25*
median (min-max)	1,8 (0,8-4,9)	*2,4 (1,2-4,4)*
Baseline creatinine, 11-19 months after biopsy		
n	75	*21*
median (min-max)	1,8 (0,8-5,4)	*2,2 (1,2-9,5)*
Donor specific antibodies		
yes	24 (27%)	6 (21%)
no	40 (44 %)	15 (54 %)
possibly	7 (8%)	3 (11%)
no information	19 (18%)	4 (14%)
Immunosuppressive medication		
single	0 (0%)	1 (4%)
dual	7 (8%)	2 (7 %)
triple	82 (91%)	25 (89%)
quadruple	1 (1%)	0 (0%)
Renal disease		
alport syndrome	1 (1%)	1 (4%)
amyloidosis	1 (1%)	0 (0%)
diabetic nephropathy	7 (8%)	4 (14%)
glomerulopathies	39 (43%)	9 (32%)
GPA	1 (1%)	0 (0%)
HUS	0 (0%)	2 (7%)
hypertensive nephropathy	5 (6%)	1 (4%)
interstitial nephritis	1 (1%)	0 (0%)
nephrotic syndrome	3 (3%)	0 (0%)
polycystic kidney disease	7 (8%)	4 (14%)
reflux nephropathy	2 (2%)	1 (4%)
lupus nephritis	1 (1%)	1 (4%)
others	22 (24%)	5 (18%)
Histology		
Banff diagnosis		
non-rejection	42 (47%)	13 (46%)
ABMR	11 (12%)	1 (4%)
TCMR	25 (28%)	10 (36 %)
mixed rejection	10 (11%)	3 (11 %)
unclear	2 (2%)	1 (4%)
IFTA		
none	*16 (18%)*	*5 (18%)*
low	*51 (57%)*	*8 (29%)*
medium	*15 (17%)*	*4 (14%)*
high	*4 (4%)*	*10 (36%)*
unclear	*4 (4%)*	*1 (4%)*
Banff i		
n	66	*17*
0	15 (23%)	*2 (12%)*
1	44 (67%)	*6 (35%)*
2	5 (8%)	*5 (29%)*
3	2 (3%)	*4 (24%)*
Banff g		
n	66	*17*
0	48 (73%)	*15 (88%)*
1	7 (11%)	*1 (6%)*
2	9 (14%)	*1 (6%)*
3	2 (3%)	*0 (0%)*
Banff ptc		
n	66	*16*
0	47 (71%)	*11 (69%)*
1	17 (26%)	*5 (31%)*
2	2 (3%)	*0 (0%)*
3	0 (0%)	*0 (0%)*
Banff ct		
n	47	*13*
0	4 (9%)	*0 (0%)*
1	31 (66%)	*5 (38%)*
2	12 (26%)	*4 (31%)*
3	0 (0%)	*4 (31%)*
Banff ci		
n	47	*13*
0	4 (9%)	*0 (0%)*
1	30 (64%)	*5 (38%)*
2	13 (28%)	*4 (31%)*
3	0 (0%)	*4 (31%)*

**Table 2:** ROC analysis to show the discriminative ability of each parameter to identify graft deterioration (0.5 mg/dl increase of creatinine) 2–8 months and 11–19 months after the biopsy.

ROC	8 months prediction			19 months prediction		
	AUC	95% CI	P value	AUC	95% CI	P value
IFTA (Banff)	0.88	0.73 - 1.00	**0.001**	0.61	0.33 - 0.89	0.40
LOXL2+ cells	0.80	0.62 - 0.98	**0.01**	0.84	0.69 - 0.996	**0.009**
time since transplantation	0.75	0.58 - 0.92	**0.03**	0.51	0.26 - 0.77	0.91
DM/HTN	0.65	0.41 - 0.89	0.20	0.52	0.25 - 0.78	0.87

Only non-rejection biopsies. p < 0.05 are bold. CI, confidence interval. OR, Odds ratio. DM/HTN, diabetes or hypertension.

### 2.8 Multiplexed Ab-seq single cell RNA sequencing

The detailed protocol is outlined in **Supplementary Data 2**. Briefly, the biopsy was digested using DNAse I (10 mg/ml) and Collagenase I (100 U/µl) at 350 rpm and 37°C for 30 min. Fc receptors were blocked by Human TruStain FcX (Biolegend) for 5 min at room temperature. The BD Rhapsody Express System (BD Biosciences, 633707) was used for single cell RNA sequencing according to the manufacturer’s protocol.

### 2.9 Sequencing data analysis

Demultiplexing and preprocessing of the data was conducted with the BD Rhapsody Sequence Analysis Pipeline v.1.11 from SevenBridges according to the manufacturer’s protocol. After aligning the data against the human reference target hg38 and a set of Abseq antibody sequences, error-corrected count matrices were created according to the BD standard workflow.

For the import into Seurat v4.0.5 (12), the scRNA count matrices were split into two parts using R v4.0.5, separating the Abseq antibody data from the regular mRNA gene counts. The minimum number of features per cell was set to five, while the minimum number of cells for any feature to be considered was kept at 10. Multiplets were defined as cells with a very high RNA count value, and filtered accordingly. The multiplet rate was between 2.58 and 5.56%.

Abseq and mRNA count data was integrated separately for all samples with Seurat’s anchor-based integration and SCTransform function. Afterwards, the mRNA and Abseq data was merged and subsequently integrated following Seurat’s weighted nearest neighbor analysis workflow. The cell cluster plots therefore show wnnUMAP as X and Y axis. The cluster resolution was set to 0.5, resulting in 19 clusters. Differentially expressed genes per cluster were calculated using the MAST algorithm (13), and cluster annotation was performed using patterns of differentially expressed genes.

A gene set score was created for a list of ECM genes as previously reported (14). There were 20 overlapping genes in our data set that were used to calculate the ECM score (FN1, IGFBP5, THBS1, THBS2, TNC, SLIT3, MGP, LAMA2, SPP1, TGFBI, DCN, OGN, COL7A1, COL6A1, COL4A1, COL5A1, COL14A1, COL1A1, COL1A2, COL3A1). All cells were split in terciles based on the ECM score (high, medium, low).

The public scRNA dataset of Kuppe et al. (14) was analyzed using Seurat’s SCTransform integration (min.cells = 3, min.features = 50) and a maximum RNA count of 30.000 for multiplet filtering. Original annotation data were imported per cell and combined at the cluster level. Differentially expressed genes per cluster were calculated to confirm the annotated cell types of the original publication.

The gene pathway analysis was calculated using Metascape (15). It is based on published GO terms/KEGG and Reactome pathways, and identifies gene sets that are more enriched in an input gene list than expected by chance (hypergeometric test and Benjamini-Hochberg P-value correction). Our input list included the differentially expressed genes of the scRNAseq cluster 12.

## 3 Results

### 3.1 Phenotyping LOXL2-positive cells in the kidney allograft

**Figure 1:**
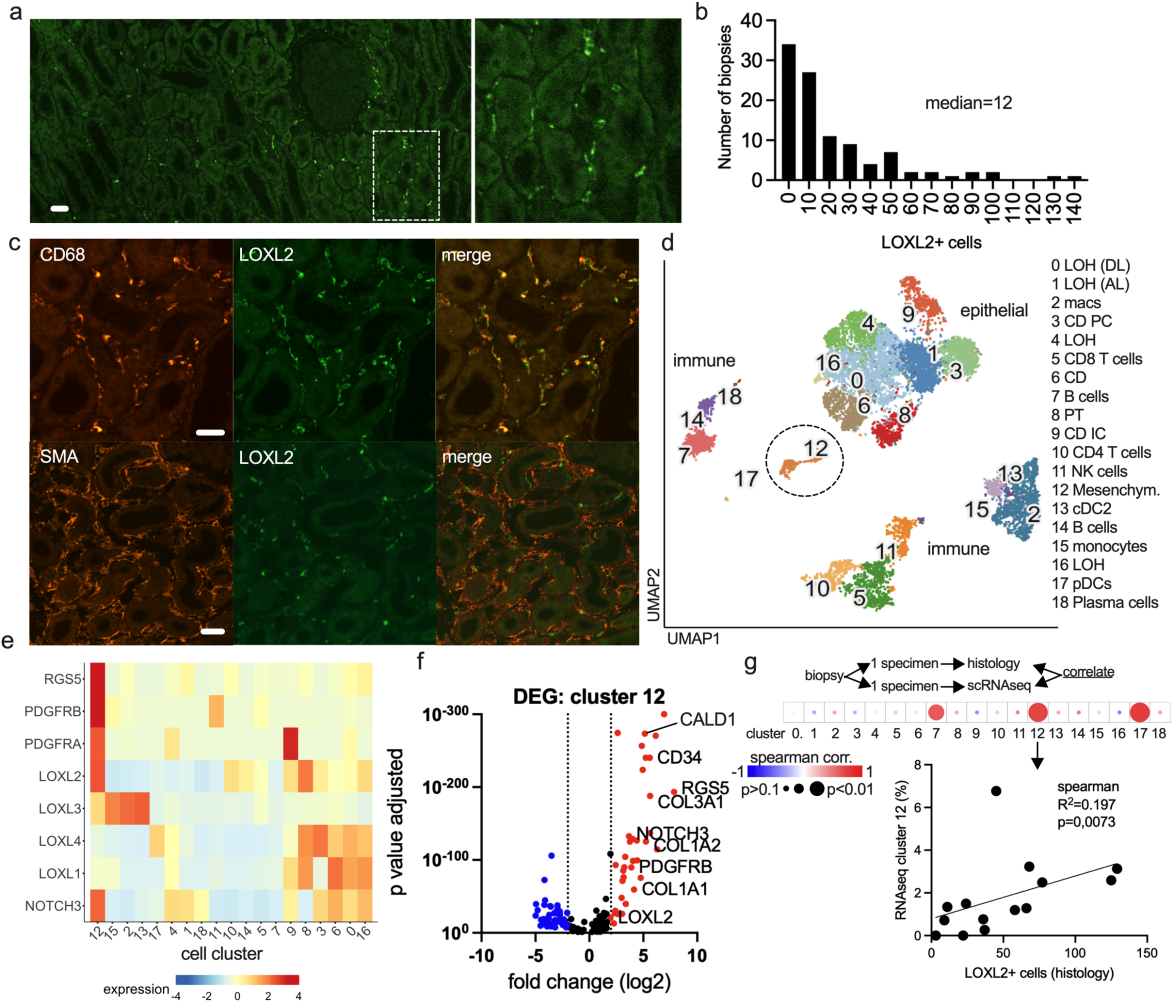
LOXL2 labels intertubular SMA-negative myofibroblasts in human kidney allografts. **(A)** Immunofluorescence staining of LOXL2 in a kidney transplant biopsy with a close up view. Scale bar 40 μm. **(B)** Relative frequency histogram of LOXL2+ cell numbers in 103 biopsies (graft age > 28 days). Patient characteristics are detailed in **Table 3**. **(C)** LOXL2 co-stainings with CD68 and smooth muscle actin (SMA). Scale bar 20 μm. **(D)** UMAP plot of cell clusters and annotations determined by single cell RNA sequencing of 18 kidney transplant biopsies. Details in Supplementary data 4. The highest LOXL2 expression is found in cluster 12 as also shown in **(E)** Heatmap of fibroblast/pericyte-associated genes and LOXL isoforms in all cell clusters. **(F)** Differentially expressed genes (DEG) of cluster 12 are shown as volcano plot with up (red) and downregulated (blue) genes compared to all other clusters. Full list in Supplementary data 5. **(G)** Spearman correlation between LOXL2+ myofibroblasts (histology) and cell cluster numbers based on the scRNAseq analysis. Color indicates the correlation coefficient and dot size the significance. The cluster 12 correlation is shown in detail.

LOXL2 staining in human kidney allografts primarily labeled intertubular cells characterized by extended cell bodies and dendritic-like extensions (**Figure 1A**). Most cells were located in the intertubular and perivascular interstitium (**Figure 1A**; **Supplementary Data 3**). We also used a different anti-LOXL2 antibody to validate the staining pattern (see methods). To quantify LOXL2+ cells, immunohistochemistry was performed on indication biopsies from 118 patients (**Table 3**). 34% of the biopsies contained low numbers of LOXL2+ cells (0–10 per field of view (FOV)). The maximum was 140 cells/FOV (**Figure 1B**). Co-staining analyses revealed that these LOXL2+ cells expressed CD68 but did not express smooth muscle actin (SMA), PDGFRβ, CD45, MHC-II or CXCL12 (**Figure 1C**; **Supplementary Data 3**), suggesting a non-leukocyte origin. To corroborate these findings, we performed single-cell RNA sequencing (scRNA-seq) using a targeted gene panel comprising approximately 500 immune- and kidney-related genes. This analysis included 18 indication biopsies, covering both non-rejection and rejection cases, and resulted in the identification of 19 distinct cell clusters (**Figure 1D**; **Supplementary Data 4**). The highest expression of LOXL2 RNA was observed in cluster 12 (**Figure 1E**), which was identified as (myo)fibroblasts or activated pericytes based on the differentially expressed (DE) genes (**Figure 1F**). They included classical pericyte genes (RGS5, PDGFRB, NOTCH3, MGP, HEG1, NRP1, CD34), activation/inflammation markers (IL33, CCL2, TNFSF10, SOCS3, CD40, LGALS1/9) and matrix-associated genes (COL1A1, COL1A2, COL3A1, COL14A1, FN1, LOXL2, TGFBI, MGP) (**Supplementary Data 5**). Notably, the LOXL1, LOXL3, and LOXL4 isoforms were not enriched in this cluster. For 15 of these biopsies, a second specimen was examined to assess the abundance of LOXL2+ cells using microscopy. The analysis revealed a significant correlation between the LOXL2+ cell counts observed in histology and the scRNA-seq-defined cluster 12 (**Figure 1G**) linking manual cell counts to a specific cell type. There was also an association between LOXL2+ cells and B cells and plasmacytoid dendritic cells, corresponding to clusters 7 and 17, respectively (**Figure 1G**).

**Table 3:** Multivariable and univariate logistic regression analyses showing independent predictors of graft deterioration (0.5mg/dl increase of creatinine) 2–8 months and 11–19 months after the biopsy.

multivariate	8 months prediction			19 months prediction		
	OR	95% CI	P value	OR	95% CI	P value
IFTA (Banff)	10.4	1.63 - 222.1	**0.01**	1.61	0.24 to 11.61	0.60
LOXL2+ cells	1.03	0.98 - 1.11	0.20	1.06	1.01 to 1.15	**0.013**
time since transplantation	1.00	0.99 - 1.00	0.40	0.99	0.99 to 1.00	0.33
DM/HTN	25.0	1.22 - 1977	**0.03**	5.05	0.17 to 128.4	0.31

univariate	OR	95% CI	p value	OR	95% CI	p value
IFTA (Banff)	15.8	3.48 - 166	**0.0001**	2.37	0.65 - 10.52	0.18
LOXL2+ cells	1.04	1.00 - 1.08	**0.02**	1.52	1.01 - 1.11	**0.008**
time since transplantation	1.00	1.00 - 1.001	**0.02**	0.99	0.99 - 1.00	0.82
DM/HTN	7.5	1.01 - 69.8	**0.049**	1.4	0.06 - 13.97	0.79

Only non-rejection biopsies. Odds ratios (OR) >1 indicate increased risk. p-values (Wald test), α, 0.05. p < 0.05 are bold. CI, confidence interval. OR, Odds ratio. DM/HTN, diabetes or hypertension.

**Figure 2:**
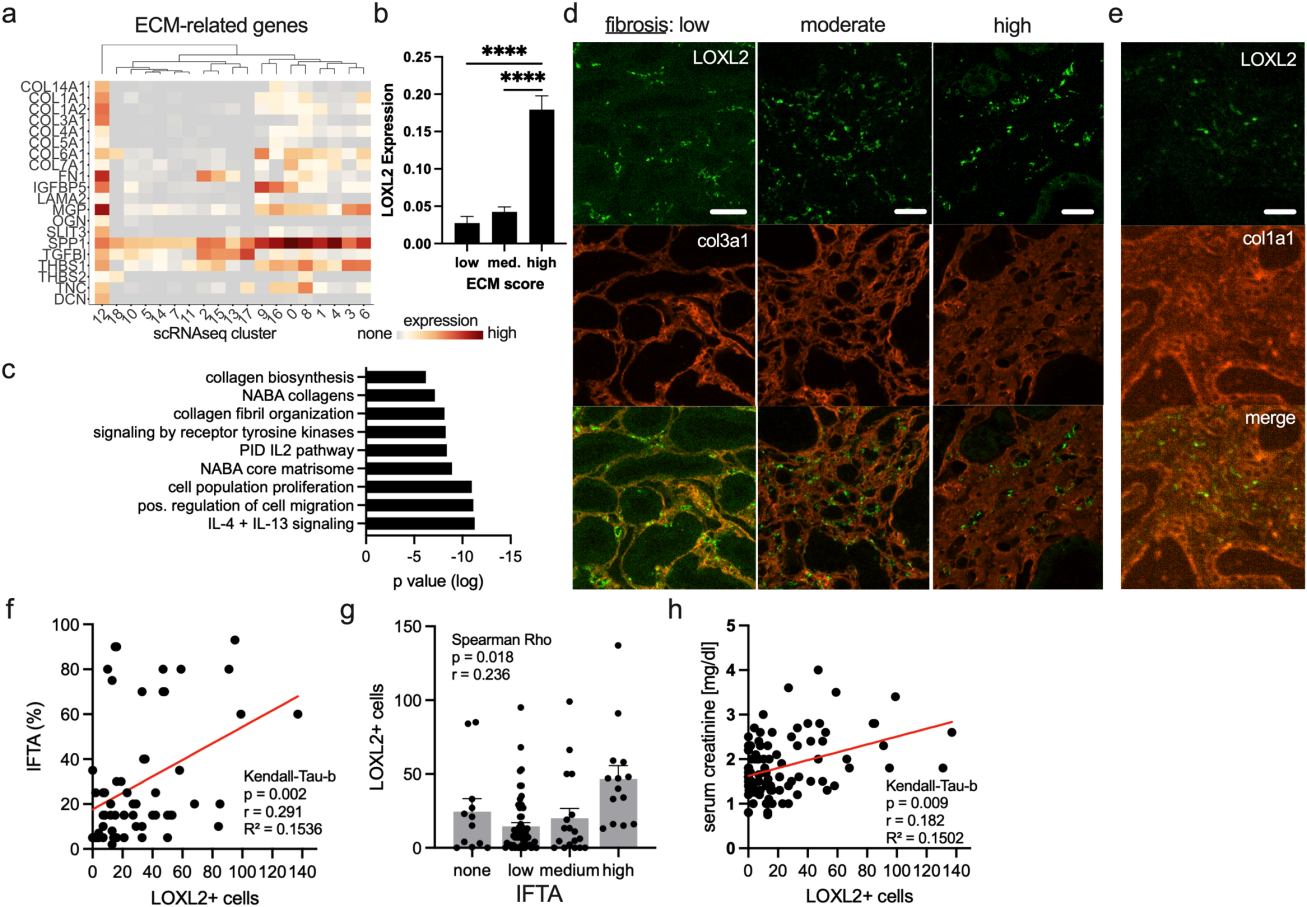
LOXL2-positive myofibroblasts show high ECM activity, and populate fibrotic and non-fibrotic areas. **(A–C)** are related to the scRNAseq data in **Figure 1**. **(A)** Expression of ECM-related genes are visualized across all cell clusters. **(B)** A cumulative ECM score was calculated and cells were divided in low, medium and high ECM groups. The average LOXL2 expression in these groups is shown. Unpaired t-test. ****p < 0.0001 **(C)** Gene pathway enrichment analysis was performed using the DE-genes of cluster 12. **(D, E)** Representative regions with low, moderate and high degrees of fibrosis are shown stained with anti-collagen 3 or 1. Scale bar 20 μm. **(F, G, H)** Fibrosis as percentage and as Banff category 0–3 as well as serum creatinine values were plotted against the number of LOXL2+ cells. A linear regression curve is shown. Kendall Tau and Spearman Rho correlation analysis was performed. Each dot represents one biopsy.

We used a predefined list of genes (14) to calculate an extracellular matrix (ECM) score for each cell of the scRNA-seq data set. The highest expression was found in cluster 12 (**Figure 2A**). Cells with the highest ECM scores also had the highest levels of LOXL2 gene expression (**Figure 2B**). Gene pathway enrichment analysis further highlighted numerous ECM-related pathways, along with interleukin-4 and interleukin-13 signaling (**Figure 2C**). Given the strong ECM signature in cluster 12, the intertubular localization, the gene enrichments and staining patterns, we infer that these cells were a LOXL2+ SMA- CD68+ myofibroblast subset.

These cells could be detected in allografts with low, moderate and high degrees of fibrosis including collagen 3 and 1 (**Figure 2D**). Notably, they consistently localized in collagen 3-free gaps, a pattern not observed with collagen 1 (**Figures 2D, E**). Using the data set with 118 biopsies, there was a significant positive correlation between LOXL2+ cells and the percentage of fibrosis (Kendall Tau, p=0.002, **Figure 2F**), as well as a significant association with the Banff IFTA score (interstitial fibrosis and tubular atrophy. Spearman Rho, p=0.018, **Figure 2G**). Consistent with these findings, serum creatinine, a marker of allograft function, also showed a significant correlation with LOXL2+ cell counts (Kendall Tau, p=0.009, **Figure 2H**).

### 3.2 LOXL2-positive cells in native kidneys

**Figure 3:**
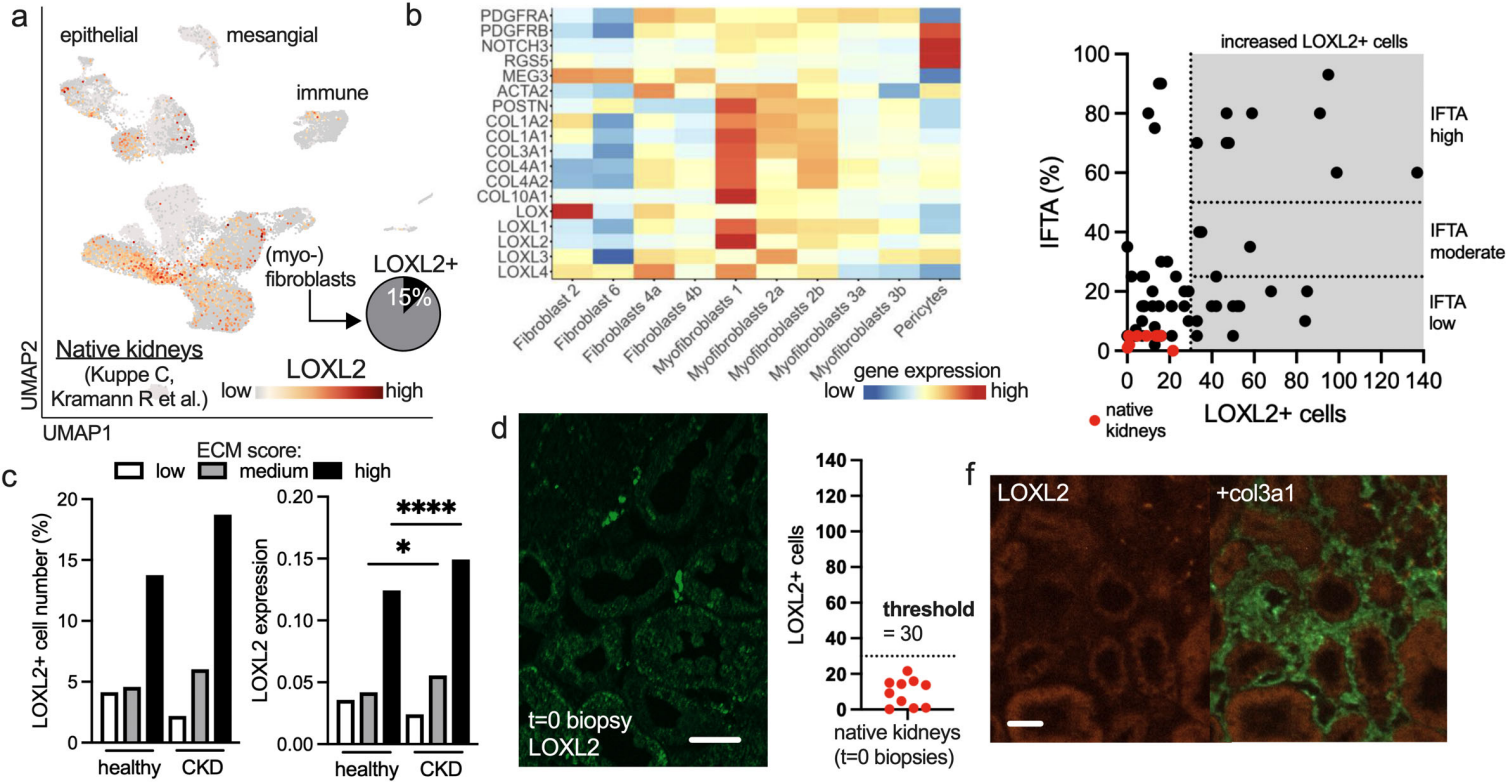
Naive kidneys contain low amounts of LOXL2-positive myofibroblasts. Panels a-c refer to published data of native human kidneys with and without chronic kidney disease (14). **(A)** UMAP plot of single cell sequencing data of a subset of 53,672 CD10 (tubulus)-negative cells (*n* = 7 eGFR > 60; *n* = 4 eGFR < 60 ml/min). The LOXL2 expression is color-coded. LOXL2 RNA expression is detectable in 15% of all myofibroblasts. **(B)** ECM-related genes are shown in a heatmap across all (myo)fibroblast subsets. **(C)** A cumulative ECM score was calculated and the LOXL2+ cell number and expression was plotted in healthy and CKD kidneys. **(D)** Representative LOXL2 staining of a native kidney right before transplantation (t=0 biopsy). Scale bar 20 μm. Analysis of 10 t=0 biopsies shows a median of 11 and a maximum of 21 LOXL2+ cells/FOV. A threshold of 30 cells/FOV was defined to identify pathologically elevated LOXL2+ cell numbers (see methods). **(E)** LOXL2+ cells versus fibrosis (%) plot of t=0 biopsies (red) together with transplant biopsies (black). The LOXL2 threshold of 30 cells is highlighted as well as the Banff IFTA definitions of low (<25%), moderate (25-50%) and high fibrosis (>50%). **(F)** Representative picture of a fibrotic region without the presence of LOXL2+ myofibroblasts in a transplant kidney. Scale bar 30 μm.

We next investigated whether native kidneys also harbor LOXL2+ myofibroblasts. In a previously published single-cell sequencing dataset of human kidneys with and without chronic kidney disease (CKD), we observed that the LOXL2 gene was predominantly enriched in myofibroblast populations (**Figure 3A**). Approximately 15% of all myofibroblasts expressed LOXL2, and this expression largely attributed to a specific subpopulation distinct from pericytes (**Figure 3B**). We repeated the ECM score analysis and confirmed that LOXL2+ myofibroblasts are characterized by the highest ECM signature (**Figure 3C**). This signature was evident in healthy kidneys and slightly elevated in CKD (**Figure 3C**).

We then analyzed t=0 biopsies, i.e. biopsies of native kidney grafts taken immediately before transplantation, and detected low numbers of intertubular LOXL2+ cells (**Figure 3D**). The cell count never exceeded 21 and the mean was 9 cells/FOV (n=10, **Figure 3D**). Based on this data and a ROC analysis prediction (see also method section), we established a threshold of 30 LOXL2+ cells/FOV to indicate abnormally high LOXL2 cell numbers in kidney biopsies (**Figure 3D**). When we compared these t=0 biopsies to all other transplant biopsies using the 30-cell threshold, we found elevated levels of LOXL2+ myofibroblasts across all IFTA groups—low, moderate, and high (**Figure 3E**). Interestingly, there were cases of low IFTA with high LOXL2+ cell numbers, and high IFTA with low LOXL2+ cell counts. The latter could also be corroborated in histological analysis (**Figure 3F**).

### 3.3 Rapid appearance of LOXL2-positive cells in inflammation

**Figure 4:**
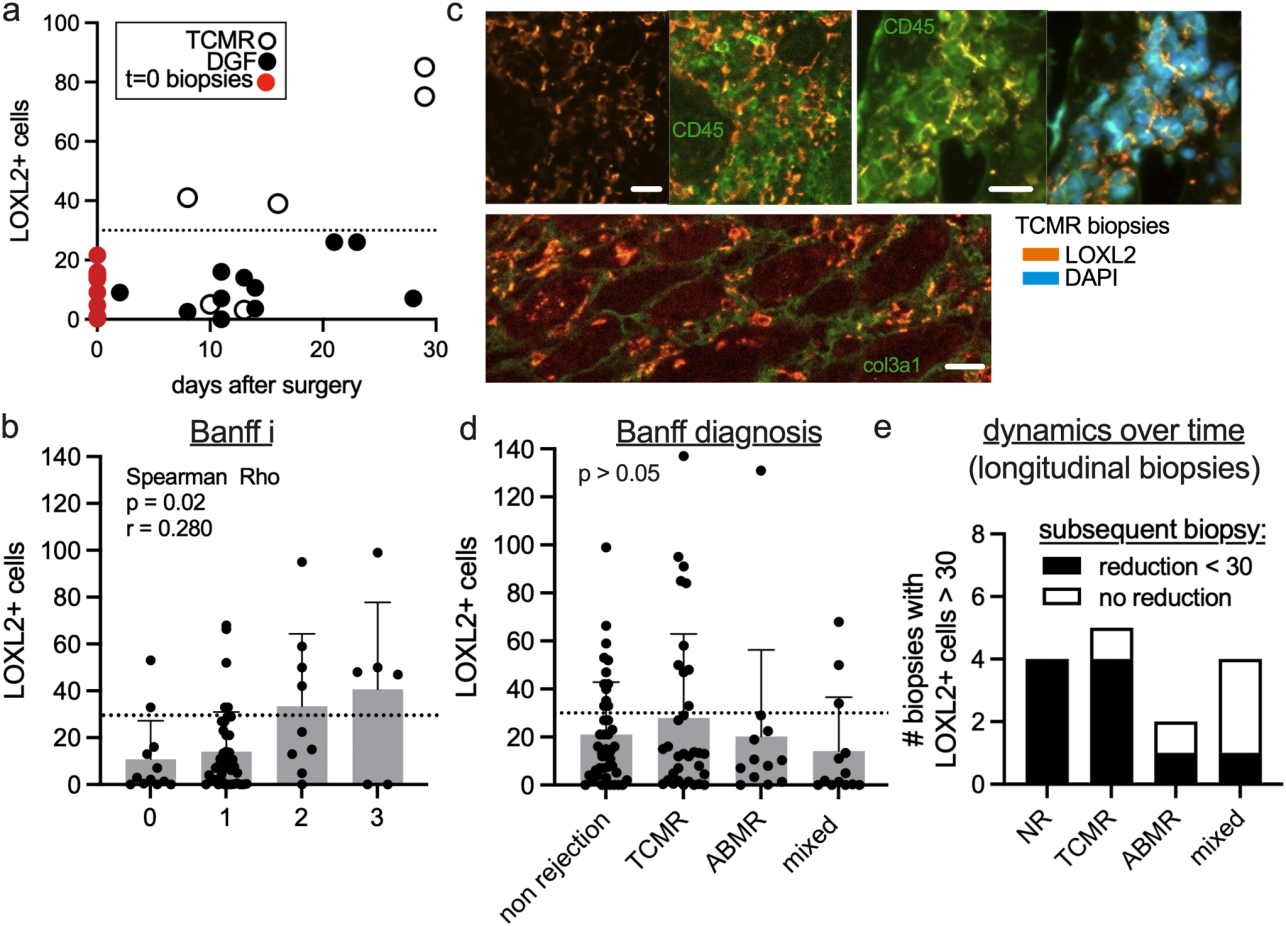
Inflammation can trigger the fast appearance of LOXL2-positive myofibroblasts. **(A)** The LOXL2+ cell numbers were counted in biopsies <= 30 days after transplant surgery. The pathologic threshold of 30 is marked as a dotted line and t=0 biopsies are included as red dots for reference (see **Figure 3**). Open and closed circles represent TCMR and delayed graft function (DGF), respectively. **(B)** Correlation of the Banff i score (inflammation 0-3) with LOXL2+ cell numbers. Spearman Rho test. **(C)** TCMR biopsies with co-stainings of LOXL2 and the leukocyte marker CD45 (green). DAPI as a nuclear marker shows cell aggregates. Co-staining with collagen 3 shows high LOXL2+ myofibroblast density in areas of low fibrosis. Scale bars 20 μm. **(D)** Association of the Banff diagnosis with LOXL2+ cell numbers. Kruskal-Wallis test. **(E)** Patients with sequential indication biopsies were screened for LOXL2+ cell numbers > 30 (pathological threshold), and the subsequent biopsy was evaluated whether a reduction below the threshold of 30 cells occurred. NR = non rejection.

Given that LOXL2+ myofibroblasts are low in native kidneys but significantly elevated in some allografts, we examined the temporal dynamics of their appearance. In a subcohort of biopsies taken within the first 28 days after transplant surgery (n=15), we observed a notable increase in LOXL2+ cells as early as 9 days post-surgery (**Figure 4A**). Notably, all biopsies with elevated LOXL2+ cells were associated with T-cell-mediated rejection (TCMR), while those diagnosed with delayed graft function (DGF) had LOXL2+ cell counts within the normal range (**Figure 4A**). The Banff inflammation score (i) showed a significant correlation with the number of LOXL2+ cells (Spearman Rho, p=0.02, **Figure 4B**). Immunofluorescence imaging of TCMR biopsies revealed that LOXL2+ cells were frequently enriched and often found within leukocyte aggregates (**Figure 4C**). In some biopsies, the presence of high LOXL2+ myofibroblast numbers was associated with low levels of fibrosis (**Figure 4C**, bottom panel; **Figure 3E**). No significant association was observed between LOXL2+ cell counts and specific types of rejection or other Banff criteria (**Figure 4D**; **Table 3**).

As LOXL2+ myofibroblasts rapidly appeared during graft inflammation, we asked if they can also decrease, i.e. following immunosuppressive treatment. To address this, we analyzed a kidney graft dataset comprising sequential indication biopsies from the same patients (8). We specifically filtered the biopsies for elevated LOXL2+ cell counts (≥ 30) and then assessed whether subsequent biopsies from the same allografts exhibited a reduction in LOXL2+ cells below this threshold (< 30). Our analysis revealed that LOXL2+ cell counts frequently decreased in subsequent biopsies, regardless of the rejection or non-rejection status (**Figure 4E**). It is noteworthy that in one follow-up biopsy, despite the resolution of rejection, LOXL2+ myofibroblast levels remained pathologically elevated.

### 3.4 LOXL2-positive cells as predictors of graft dysfunction

**Figure 5:**
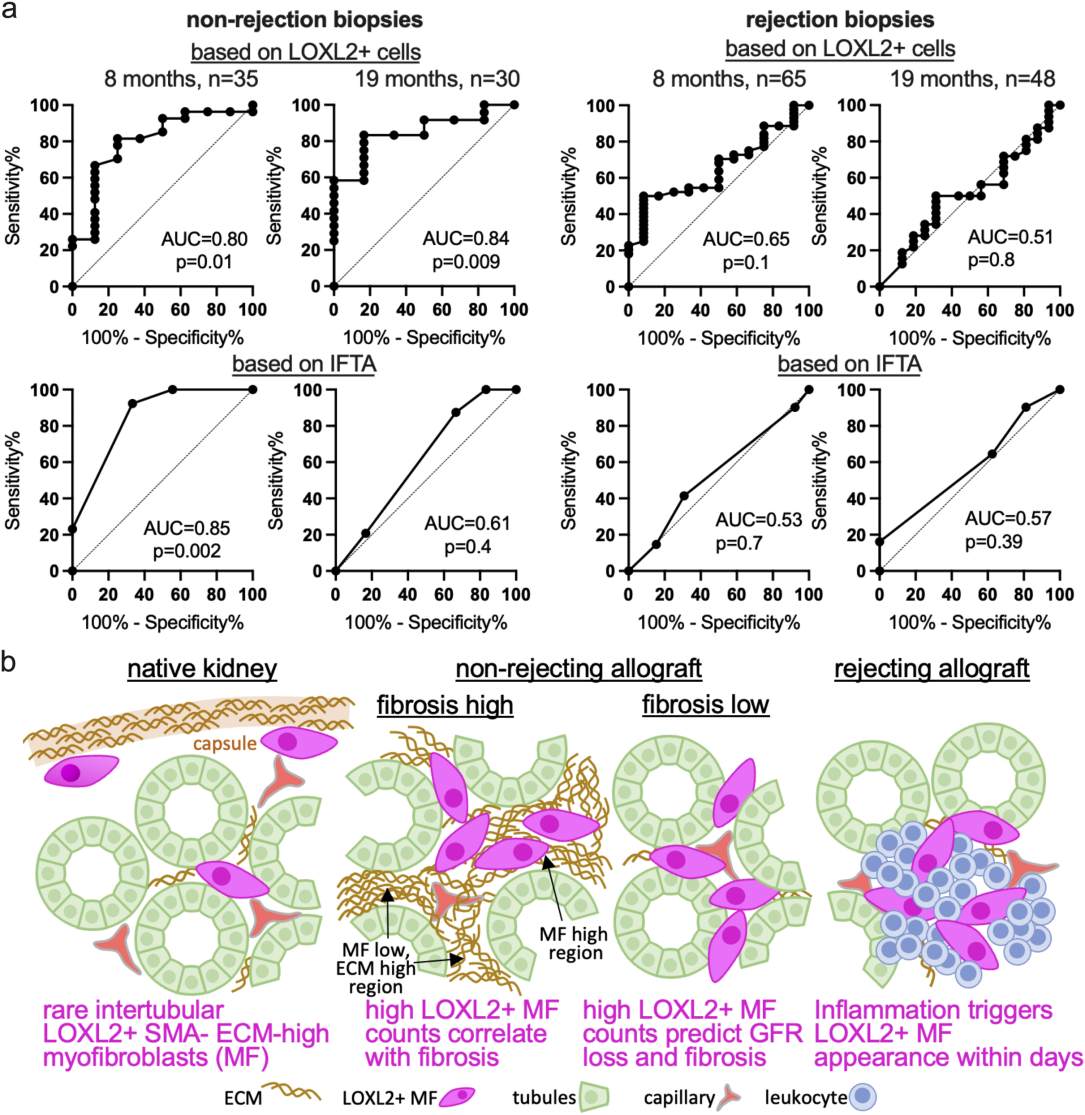
Prediction of the long-term graft function. **(A)** Performance of LOXL2+ cell counts (top) or IFTA (bottom) to predict a serum creatinine increase in 2–8 months and 11–19 months after the biopsy using ROC curves. Details are outlined in the method and result section, and in **Table 1**. The analysis was performed separately for non-rejecting (left) and rejecting (right) biopsies. AUC, area under the curve. **(B)** Summary of the research findings highlighting the roles of LOXL2+ myofibroblasts in rejecting and non-rejecting kidney allografts. ECM, extracellular matrix; SMA, smooth muscle actin; MF, myofibroblast; GFR, glomerular filtration rate.

Since LOXL2+ cells bear high ECM signatures, we hypothesized that elevated counts could indicate an increased risk of developing allograft dysfunction. We have previously shown that the number of LOXL2 cells in the biopsy correlates with the development of fibrosis (8). Now we tested whether it could also predict functional outcomes at two follow-up time points - up to 8 months and up to 19 months after the biopsy. A logistic regression model including time since transplantation, IFTA, hypertension/diabetes status (HTN/DM), and mean LOXL2+ cells in the biopsy was applied in non rejection biopsies to predict a 0.5 mg/dl increase of the baseline creatinine (11). As expected, IFTA and the HTN/DM were independent and strong predictors of the outcome at 8 months (**Table 2**). Here, LOXL2 showed univariate association, but not after multivariate adjustment. LOXL2 cell counts were the only significant predictor at the 19 months time point using both the multivariate and univariate approach (**Table 2**). In all cases, the Odds ratios of LOXL2 cell counts were small and the ROC analysis shows high discriminative ability (**Table 1**; **Figure 5**). For long-term prediction, LOXL2+ cells outperformed the IFTA score (AUC = 0.84, p=0.009 for LOXL2+ cells vs. AUC = 0.61, p=0.4 for IFTA). The same analysis in biopsies with rejection showed no significant results in all conditions tested (**Figure 5**).

## 4 Discussion

Myofibroblasts are a hallmark of organ fibrosis (16, 17). Smooth muscle actin (SMA) has been used as the standard marker in many studies. However, single cell technologies demonstrated a more complicated and diverse landscape of tissue resident myofibroblasts (18), spanning from fibroblast- to pericyte- und tubulus-like signatures (14, 19, 20). The biological and clinical significance of many of these subsets remains largely unknown.

Our analysis suggests LOXL2+ as a marker for a renal SMA-negative myofibroblast subset. The co-staining of LOXL2 with CD45, a leukocyte marker, was negative, and single-cell sequencing data from allograft biopsies indicated a mesenchymal origin. The LOXL2+ scRNA-seq cell cluster 12 was defined by a pericyte- and fibroblast-like signature with high extracellular matrix scores. Among the most highly differentially expressed genes in this cluster was PDGFRβ, NOTCH3, and RGS5, with the latter being predominantly associated with pericytes and myofibroblasts (21). Despite the transcriptomic similarities to pericytes (22), the spatial distribution of LOXL2+ cells was mostly intertubular rather than strictly perivascular. This was most evident in specimen with high LOXL2+ numbers such as nephrectomy samples. Additionally, when analyzing published RNA-seq data from native kidneys, LOXL2 gene expression was specific to myofibroblasts and largely absent in pericytes (14). Here, several different myofibroblast cell populations featured significantly elevated LOXL2 gene expressions (14, 23, 24). Periostin (POSTN) has been proposed as a key gene defining distinct myofibroblasts in both the kidney (14) and the heart (25). We showed in native kidney that the LOXL2+ myofibroblast subtype exhibits the highest expression of POSTN, while the expression of ACTA2 (which encodes SMA) was comparatively lower than in other fibroblast and myofibroblast subsets. It is also conceivable that LOXL2+ cells are involved in the process of macrophage (CD68+) to myofibroblast (26) or pericyte to myofibroblast (27) transition. In the infarcted heart, pericytes acquire a fibrogenic phenotype with upregulated LOXL2 expression (28). It is important to note that due to the low number of cells in the biopsies, the sequencing analysis identified only one mesenchymal cell cluster. Thus, fibroblast subpopulations and transition phenotypes may be underrepresented in our dataset. Larger studies are required with higher cell numbers per biopsy and a more balanced patient selection. Mechanistic analyses would be insightful to characterize their biological role during fibrogenesis.

There is ongoing debate regarding the presence and role of myofibroblasts in healthy kidneys. Some argue that myofibroblasts are absent in healthy kidneys and only emerge during pathological conditions (29). However, alternative views suggest that these cells may be part of the normal cellular landscape, contributing to physiological functions such as homeostatic matrix synthesis (30). Our data reveal the presence of LOXL2+ cells in native non-CKD kidneys, albeit in low numbers. We did not exclude the possibility of a LOXL2 positive pericyte subset in healthy kidneys. In addition, chronic low-grade inflammation which is common in many potential diseases of the graft donor such as CKD, diabetes, cardiovascular disease, and obesity (31–33) could confound the data. In native kidneys affected by CKD, we found an increase in LOXL2+ cell numbers and expression. However, the most significant elevation—up to a 20-fold increase compared to native kidneys—is observed in kidney transplants.

A notable characteristic of LOXL2+ myofibroblasts is their rapid appearance. While native kidneys harbor only low amounts of these cells, biopsies taken as early as nine days after transplant surgery show a marked increase in LOXL2+ myofibroblasts, suggesting immune-mediated mechanisms. This was further supported by our retrospective analysis, which revealed a significant correlation with tissue inflammation as defined by the Banff i score. Based on these findings, we identify “inflammation” as the primary associated phenotype driving the increase in LOXL2+ myofibroblasts, though other immune factors may also contribute. Infiltrating leukocytes frequently organize into tissue aggregates, and we show that these aggregates often contain a high density of LOXL2+ myofibroblasts. Indeed, a SPP1+ macrophage subset was recently found to orchestrate myofibroblast activation in a platelet-dependent fashion (34), demonstrating a close link between myofibroblast biology and inflammation.

The rapid kinetics and association with inflammation raises the question of the lineage origin. Previous work using RNA trajectory analyses suggested fibroblasts and pericytes as main origins of renal myofibroblasts but transitions from macrophages or tubule cells have also been described (35). LOXL2+ cells or their immediate precursors might also directly derive from the blood. This is an intriguing possibility as an infiltrating, recipient-derived fibroblast subset has been previously suggested in kidney allografts (36).

Pathologically elevated levels of LOXL2+ myofibroblasts were not irreversible. In our analysis of sequential biopsies from the same allografts, we observed that high levels of LOXL2+ cells can significantly decrease in follow-up biopsies. While the possibility of sampling errors cannot be excluded, these findings suggest that LOXL2+ inflammation-associated myofibroblasts are responsive to immunosuppression. In our study, non-rejecting grafts had a high variation of LOXL2+ myofibroblast abundance, ranging from 0 to nearly 100 cells per area. We have shown that high LOXL2+ cell numbers predict the development of interstitial fibrosis (8), and associate with a progressive decline in graft function over the following 19 months. Tissue fibrosis was the main determinant of graft deterioration in the multivariate logistic regression analysis, while LOXL2+ cells showed only small Odds ratios. Thus, interstitial fibrosis and LOXL2+ cells might be part of the same biological response. Whether there is additional value of LOXL2+ cells as a tissue biomarker requires further validation.

Together, these findings suggest LOXL2+ cells as inflammation-associated myofibroblasts in kidney allografts and a potential risk biomarker for early renal fibrogenesis.

## Data Availability

The datasets presented in this study can be found in online repositories. The names of the repository/repositories and accession number(s) can be found below: https://www.ncbi.nlm.nih.gov/geo/, GSE277703.

## References

[1] Codina S Manonelles A Tormo M Sola A Cruzado JM . Chronic kidney allograft disease: new concepts and opportunities. Front Med. (2021) 8. doi: 10.3389/fmed.2021.660334, PMID: 34336878 PMC8316649

[2] Wekerle T Segev D Lechler R Oberbauer R . Strategies for long-term preservation of kidney graft function. Lancet. (2017) 389:2152–62. doi: 10.1016/S0140-6736(17)31283-7, PMID: 28561006

[3] Li C Yang CW . The pathogenesis and treatment of chronic allograft nephropathy. Nat Rev Nephrol. (2009) 5:513–9. doi: 10.1038/nrneph.2009.113, PMID: 19636333

[4] Langewisch E Mannon RB . Chronic allograft injury. CJASN. (2021) 16:1723–9. doi: 10.2215/CJN.15590920, PMID: 33820759 PMC8729407

[5] Servais A Meas-Yedid V Noël LH Martinez F Panterne C Kreis H . Interstitial fibrosis evolution on early sequential screening renal allograft biopsies using quantitative image analysis. Am J Transplant. (2011) 11:1456–63. doi: 10.1111/j.1600-6143.2011.03594.x, PMID: 21672152

[6] Nankivell BJ Borrows RJ Fung CLS O’Connell PJ Allen RDM Chapman JR . The natural history of chronic allograft nephropathy. N Engl J Med. (2003) 349:2326–33. doi: 10.1056/NEJMoa020009, PMID: 14668458

[7] Manonelles A Guiteras R Melilli E Lazzeri E Goma M Crespo E . The presence of urinary renal progenitor cells in stable kidney transplant recipients anticipates allograft deterioration. Front Physiol. (2018) 9:1412. doi: 10.3389/fphys.2018.01412, PMID: 30364198 PMC6191504

[8] Buscher K Heitplatz B van Marck V Song J Loismann S Rixen R . Data-driven kidney transplant phenotyping as a histology-independent framework for biomarker discovery. J Am Soc Nephrol. (2021) 32:1933–45. doi: 10.1681/ASN.2020121685, PMID: 34078665 PMC8455252

[9] Poe A Martinez Yus M Wang H Santhanam L . Lysyl oxidase like-2 in fibrosis and cardiovascular disease. Am J Physiol Cell Physiol. (2023) 325:C694–707. doi: 10.1152/ajpcell.00176.2023, PMID: 37458436 PMC10635644

[10] Haghayegh S Kang HA Khoshnevis S Smolensky MH Diller KR . A comprehensive guideline for Bland-Altman and intra class correlation calculations to properly compare two methods of measurement and interpret findings. Physiol Meas. (2020) 41:055012. doi: 10.1088/1361-6579/ab86d6, PMID: 32252039

[11] Hariharan S McBride MA Cherikh WS Tolleris CB Bresnahan BA Johnson CP . Post-transplant renal function in the first year predicts long-term kidney transplant survival. Kidney Int. (2002) 62:311–8. doi: 10.1046/j.1523-1755.2002.00424.x, PMID: 12081593

[12] Hao Y Hao S Andersen-Nissen E Mauck WM Zheng S Butler A . Integrated analysis of multimodal single-cell data. Cell. (2021) 184:3573–87. doi: 10.1016/j.cell.2021.04.048, PMID: 34062119 PMC8238499

[13] Finak G McDavid A Yajima M Deng J Gersuk V Shalek AK . MAST: a flexible statistical framework for assessing transcriptional changes and characterizing heterogeneity in single-cell RNA sequencing data. Genome Biol. (2015) 16:278. doi: 10.1186/s13059-015-0844-5, PMID: 26653891 PMC4676162

[14] Kuppe C Ibrahim MM Kranz J Zhang X Ziegler S Perales-Patón J . Decoding myofibroblast origins in human kidney fibrosis. Nature. (2021) 589:281–6. doi: 10.1038/s41586-020-2941-1, PMID: 33176333 PMC7611626

[15] Zhou Y Zhou B Pache L Chang M Khodabakhshi AH Tanaseichuk O . Metascape provides a biologist-oriented resource for the analysis of systems-level datasets. Nat Commun. (2019) 10:1523. doi: 10.1038/s41467-019-09234-6, PMID: 30944313 PMC6447622

[16] Younesi FS Miller AE Barker TH Rossi FMV Hinz B . Fibroblast and myofibroblast activation in normal tissue repair and fibrosis. Nat Rev Mol Cell Biol. (2024) 25:617–38. doi: 10.1038/s41580-024-00716-0, PMID: 38589640

[17] Lurje I Gaisa NT Weiskirchen R Tacke F . Mechanisms of organ fibrosis: Emerging concepts and implications for novel treatment strategies. Mol Aspects Med. (2023) 92:101191. doi: 10.1016/j.mam.2023.101191, PMID: 37236017

[18] Buechler MB Pradhan RN Krishnamurty AT Cox C Calviello AK Wang AW . Cross-tissue organization of the fibroblast lineage. Nature. (2021) 593:575–9. doi: 10.1038/s41586-021-03549-5, PMID: 33981032

[19] Minatoguchi S Saito S Furuhashi K Sawa Y Okazaki M Shimamura Y . A novel renal perivascular mesenchymal cell subset gives rise to fibroblasts distinct from classic myofibroblasts. Sci Rep. (2022) 12:5389. doi: 10.1038/s41598-022-09331-5, PMID: 35354870 PMC8967907

[20] Shook BA Wasko RR Rivera-Gonzalez GC Salazar-Gatzimas E López-Giráldez F Dash BC . Myofibroblast proliferation and heterogeneity are supported by macrophages during skin repair. Science. (2018) 362:eaar2971. doi: 10.1126/science.aar2971, PMID: 30467144 PMC6684198

[21] Muhl L Genové G Leptidis S Liu J He L Mocci G . Single-cell analysis uncovers fibroblast heterogeneity and criteria for fibroblast and mural cell identification and discrimination. Nat Commun. (2020) 11:3953. doi: 10.1038/s41467-020-17740-1, PMID: 32769974 PMC7414220

[22] Tanaka S Portilla D Okusa MD . Role of perivascular cells in kidney homeostasis, inflammation, repair and fibrosis. Nat Rev Nephrol. (2023) 19:721–32. doi: 10.1038/s41581-023-00752-7, PMID: 37608184

[23] Gao Y Li J Cheng W Diao T Liu H Bo Y . Cross-tissue human fibroblast atlas reveals myofibroblast subtypes with distinct roles in immune modulation. Cancer Cell. (2024) 42:1764–83. doi: 10.1016/j.ccell.2024.08.020, PMID: 39303725

[24] Lake BB Menon R Winfree S Hu Q Melo Ferreira R Kalhor K . An atlas of healthy and injured cell states and niches in the human kidney. Nature. (2023) 619:585–94. doi: 10.1038/s41586-023-05769-3, PMID: 37468583 PMC10356613

[25] Kanisicak O Khalil H Ivey MJ Karch J Maliken BD Correll RN . Genetic lineage tracing defines myofibroblast origin and function in the injured heart. Nat Commun. (2016) 7:12260. doi: 10.1038/ncomms12260, PMID: 27447449 PMC5512625

[26] Wang YY Jiang H Pan J Huang XR Wang YC Huang HF . Macrophage-to-myofibroblast transition contributes to interstitial fibrosis in chronic renal allograft injury. J Am Soc Nephrol JASN. (2017) 28:2053–67. doi: 10.1681/ASN.2016050573, PMID: 28209809 PMC5491278

[27] Zhao Z Zhang Y Zhang C Zhang J Luo X Qiu Q . TGF-β promotes pericyte-myofibroblast transition in subretinal fibrosis through the Smad2/3 and Akt/mTOR pathways. Exp Mol Med. (2022) 54:673–84. doi: 10.1038/s12276-022-00778-0, PMID: 35624154 PMC9166792

[28] Alex L Tuleta I Hernandez SC Hanna A Venugopal H Astorkia M . Cardiac pericytes acquire a fibrogenic phenotype and contribute to vascular maturation after myocardial infarction. Circulation. (2023) 148:882–98. doi: 10.1161/CIRCULATIONAHA.123.064155, PMID: 37350296 PMC10527624

[29] Sato Y Yanagita M . Resident fibroblasts in the kidney: a major driver of fibrosis and inflammation. Inflammation Regen. (2017) 37:17. doi: 10.1186/s41232-017-0048-3, PMID: 29259716 PMC5725902

[30] Moeller MJ Kramann R Lammers T Hoppe B Latz E Ludwig-Portugall I . New aspects of kidney fibrosis–from mechanisms of injury to modulation of disease. Front Med. (2022) 8. doi: 10.3389/fmed.2021.814497, PMID: 35096904 PMC8790098

[31] Asghar A Sheikh N . Role of immune cells in obesity induced low grade inflammation and insulin resistance. Cell Immunol. (2017) 315:18–26. doi: 10.1016/j.cellimm.2017.03.001, PMID: 28285710

[32] Prattichizzo F De Nigris V Spiga R Mancuso E La Sala L Antonicelli R . Inflammageing and metaflammation: The yin and yang of type 2 diabetes. Ageing Res Rev. (2018) 41:1–17. doi: 10.1016/j.arr.2017.10.003, PMID: 29081381

[33] Hildebrandt X Ibrahim M Peltzer N . Cell death and inflammation during obesity: “Know my methods, WAT(son). Cell Death Differ. (2023) 30:279–92. doi: 10.1038/s41418-022-01062-4, PMID: 36175539 PMC9520110

[34] Hoeft K Schaefer GJL Kim H Schumacher D Bleckwehl T Long Q . Platelet-instructed SPP1+ macrophages drive myofibroblast activation in fibrosis in a CXCL4-dependent manner. Cell Rep. (2023) 42:112131. doi: 10.1016/j.celrep.2023.112131, PMID: 36807143 PMC9992450

[35] Wei J Xu Z Yan X . The role of the macrophage-to-myofibroblast transition in renal fibrosis. Front Immunol. (2022) 13:934377. doi: 10.3389/fimmu.2022.934377, PMID: 35990655 PMC9389037

[36] Suryawanshi H Yang H Lubetzky M Morozov P Lagman M Thareja G . Detection of infiltrating fibroblasts by single-cell transcriptomics in human kidney allografts. PloS One. (2022) 17:e0267704. doi: 10.1371/journal.pone.0267704, PMID: 35657798 PMC9165878

