# Supplementary material for: LOXL2 labels inflammation-associated myofibroblasts predicting kidney allograft dysfunction and fibrosis
Source: Front Immunol. 2026 Jan 13;16:1671117. doi: 10.3389/fimmu.2025.1671117 (PMC12834787; doi:10.3389/fimmu.2025.1671117)
Supplement: Supplementary file 1 [file DataSheet1.pdf]

Supplemental data
Schuetz P et al.
" LOXL2 labels inflammation-associated myofibroblasts predicting kidney allograft dysfunction and fibrosis."

Supplemental data 1: Antibodies used in this study

antigen	host	subtype	clone	source	cat. no.	conc.	dilution
COL1A1	mouse	IgG1	polyclonal	Cusabio	CSB-MA740543	1 mg/ml	1:20
COL3A1	goat	IgG	polyclonal	Southern Biotech	SBA-1330-01	0,4 mg/ml	1:100
PDGFRb	mouse	IgG1	PR7212	R&D	MAB1263	0,9 mg/ml	1:50
CXCL12	mouse	IgG	79018	R&D	MAB350	0,5 mg/ml	1:50
SMA	rabbit	IgG	polyclonal	Thermo Fisher	PA5-16697	0,25 mg/ml	1:200
CD68	mouse	IgG2a	514H12	Bio-Rad	MCA1815T	n/a	1:100
LOXL2	rabbit	IgG	polyclonal	Sigma	HPA-036257	0,2 mg/ml	1:200
LOXL2	rabbit	IgG	polyclonal	Abcam	ab96233	0.84 mg/mL	1:200
HLA-DR	mouse	IgG2b, k	LN3	Invitrogen	14-9956-80	0,5 mg/ml	1:200
CD45	mouse	IgG1	2B11 + PD7/26	Novus Bio	NBP2-34528	1 mg/ml	1:200

target	host	fluorophore	subtype	clone	source	cat. no.	dilution
rabbit	goat	CF™ 555	IgG(H+L)	polyclonal	Sigma Aldrich	SAB4600068	1:1000
mouse	goat	DyLight™ 488	IgG	polyclonal	Biolegend	405310	1:100
goat	donkey	Alexa Fluor 488	IgG	polyclonal	Invitrogen	A11055	1:200
mouse	goat	Alexa Fluor 647	IgG (H+L)	polyclonal	Invitrogen	A-21235	1:200

host	subtype	clone	source	cat. no.
mouse	IgG1, κ	MG1-45	Biolegend	401402
rabbit	IgG	poly29108	Biolegend	910801
goat	IgG	polyclonal	R&D	AB-108-C
mouse	IgG2b, k		Invitrogen	# 14-4732-82

Supplemental data 2: Multiplexed Ab-seq single cell RNA sequencing

The biopsy was thawed and the perirenal tissue was removed. It was then manually minced into small pieces and transferred into an 1ml tube containing 1 ml HBSS (+Ca +Mg), DNAse I (10 mg/ml) and Collagenase I (100 U/µl) for digestion at 350 rpm and 37 °C for 30 min. 2 ml PBS + 1% FCS was added to the digestion buffer followed by filtering suspensions through a 70 µm cell strainer. Samples were then centrifuged at 500g for 7 min and cell pellets were resuspended in 175 µl PBS with 1% FCS. Fc receptors were blocked by Human TruStain FcX (Biolegend) for 5 min at room temperature.

The BD Rhapsody Express System (BD Biosciences, 633707) was used for single cell RNA sequencing according to the manufacturer's protocol.The Abseq Immune Response Panel (BD Bioscience) was reconstituted in 35 µl nuclease-free water for 5 min at room temperature. 65 µl PBS/FCS was then added for a total volume of 100 µl and stored on ice until use. Per biopsy, 12.5 µl of the Abseq Immune Response Panel (30 common leukocyte markers) and 10 µl of sample tag (BD Rhapsody Multiplexing Kit) were added to the single cell suspension and incubated on ice for 30 min. After washing twice, the pellet was resuspended in 200 µl PBS/FCS. All samples were filtered through a 40 µm cell strainer and combined before centrifuged. The pellet was resuspended in 620 µl of ice-cold sample buffer. The samples were loaded onto primed nanowell cartridges (BD Biosciences, 633733), incubated at room temperature for 15 min followed by loading of cell capture beads and further incubation of 3 min at room temperature. Cells were lysed followed by Cell Capture Bead Retrieval and bead washing. Reverse transcription was performed for cDNA synthesis following Exonuclease I treatment (BD Biosciences, 633773).
Libraries were prepared using the BD Rhapsody Targeted mRNA and Abseq Amplification Kit (BD Biosciences, 633774). Targeted amplification of cDNA was performed using a human immune response panel (BD Biosciences, 633750) and a custom additional panel of 100 genes (supplemental data 6) by PCR (11-15 cycles). For double-sided DNA size selection, Agencourt AMPure XP magnetic beads (Beckman Coulter, A63880) were used to separate sample tag PCR products from mRNA target PCR products. After further PCR amplification, the PCR products were purified using Agencourt AMPure XP magnetic beads. The concentration of each sample was determined using a Qubit fluorometer and the Qubit dsDNA HS Assay Kit (Thermo Fisher Scientific, Q32851). To prepare the final libraries, the purified PCR products were indexed by PCR (6-8 cycles). The index PCR products were purified using Agencourt AMPure XP magnetic beads. Quality controls included measuring the concentration and the average fragment size of the mRNA target library and the sample tag library using the Agilent Tape Station with the High Sensitivity D1000 ScreenTape® (Agilent, 5067-5584).

The final libraries were diluted to a concentration of 4 nM and multiplexed for paired-end sequencing (150 bp) including 20% PhiX spike-in. The sequencing depth was calculated at 600, 2000 and 9000 reads/cell for the sample tag, the mRNA library and the Abseq library, respectively. Sequencing was performed using the Illumina NovaSeq 6000 sequencer at Novogene (Cambridge, UK).

Supplemental data 3: Co-expression analysis of LOXL2 and CXCL12, PDGFRb, HLA-DR (MHC-II), SMA, CD68 and CD45 by immunofluorescence microscopy using a kidney transplant nephrectomy specimen. Representative pictures are shown. Scale bar 50 um.

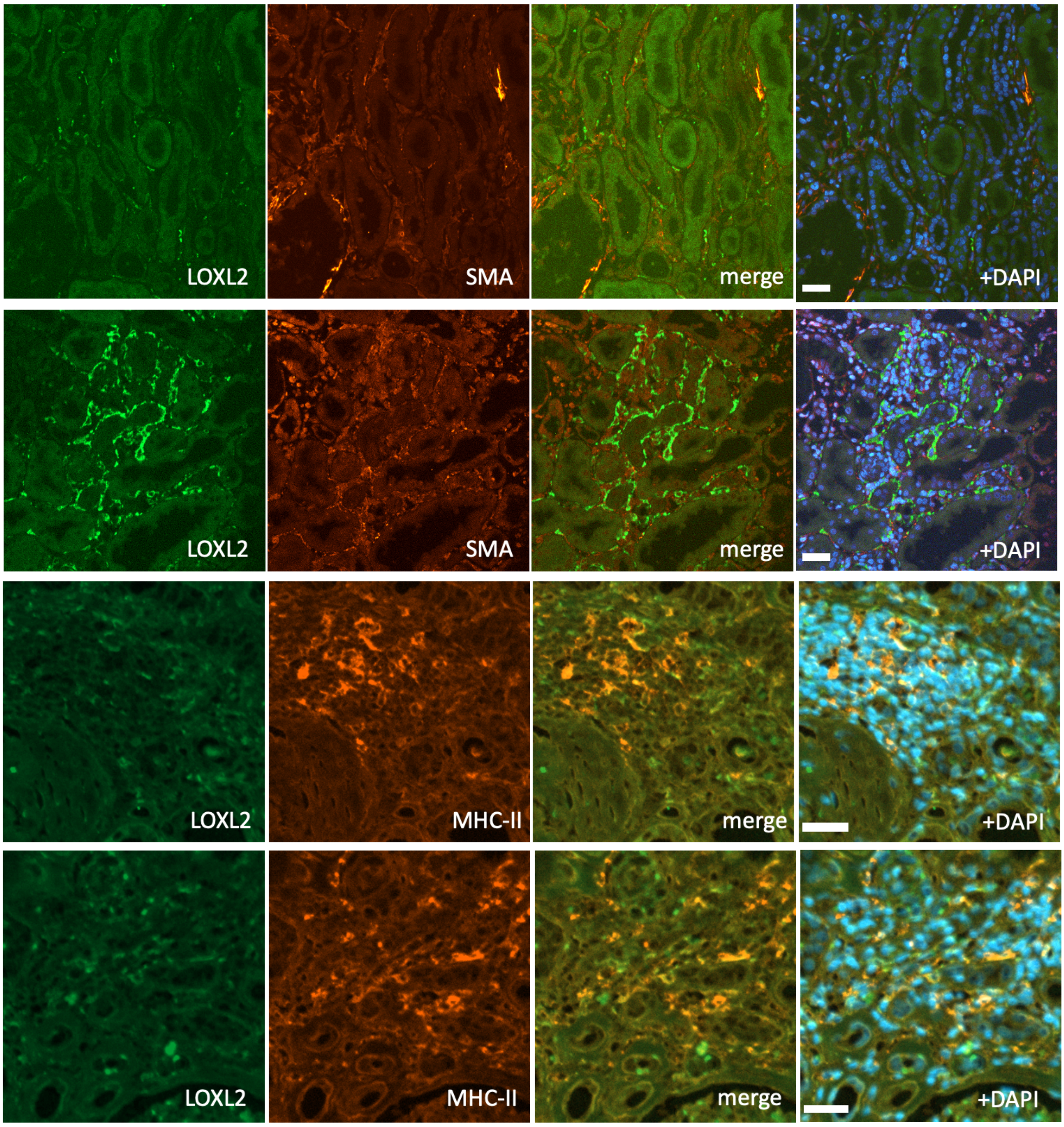

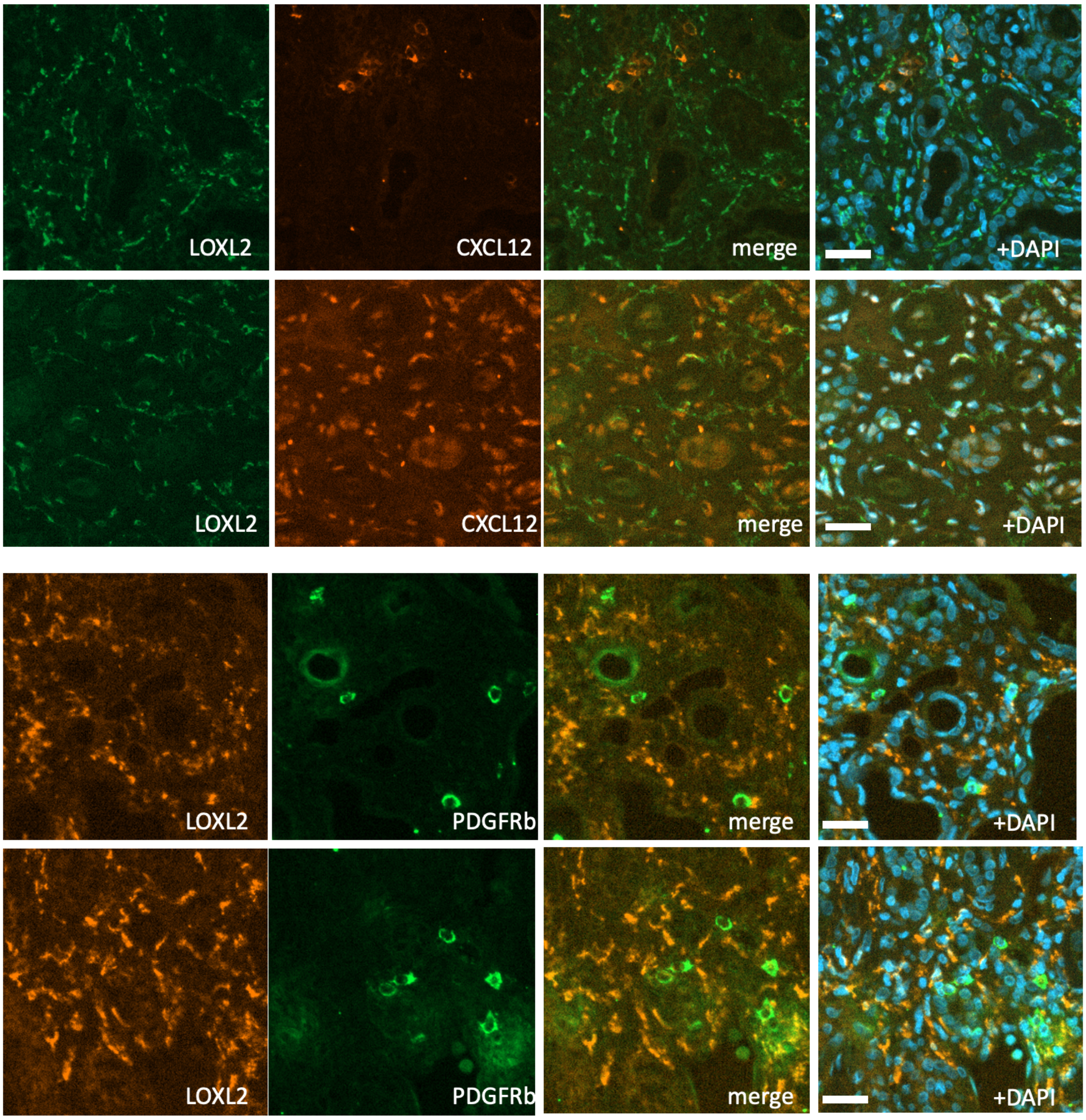
LOXL2
CXCL12
merge
+DAPI
LOXL2
CXCL12
merge
+DAPI
LOXL2
PDGFRb
merge
+DAPI
LOXL2
PDGFRb
merge
+DAPI

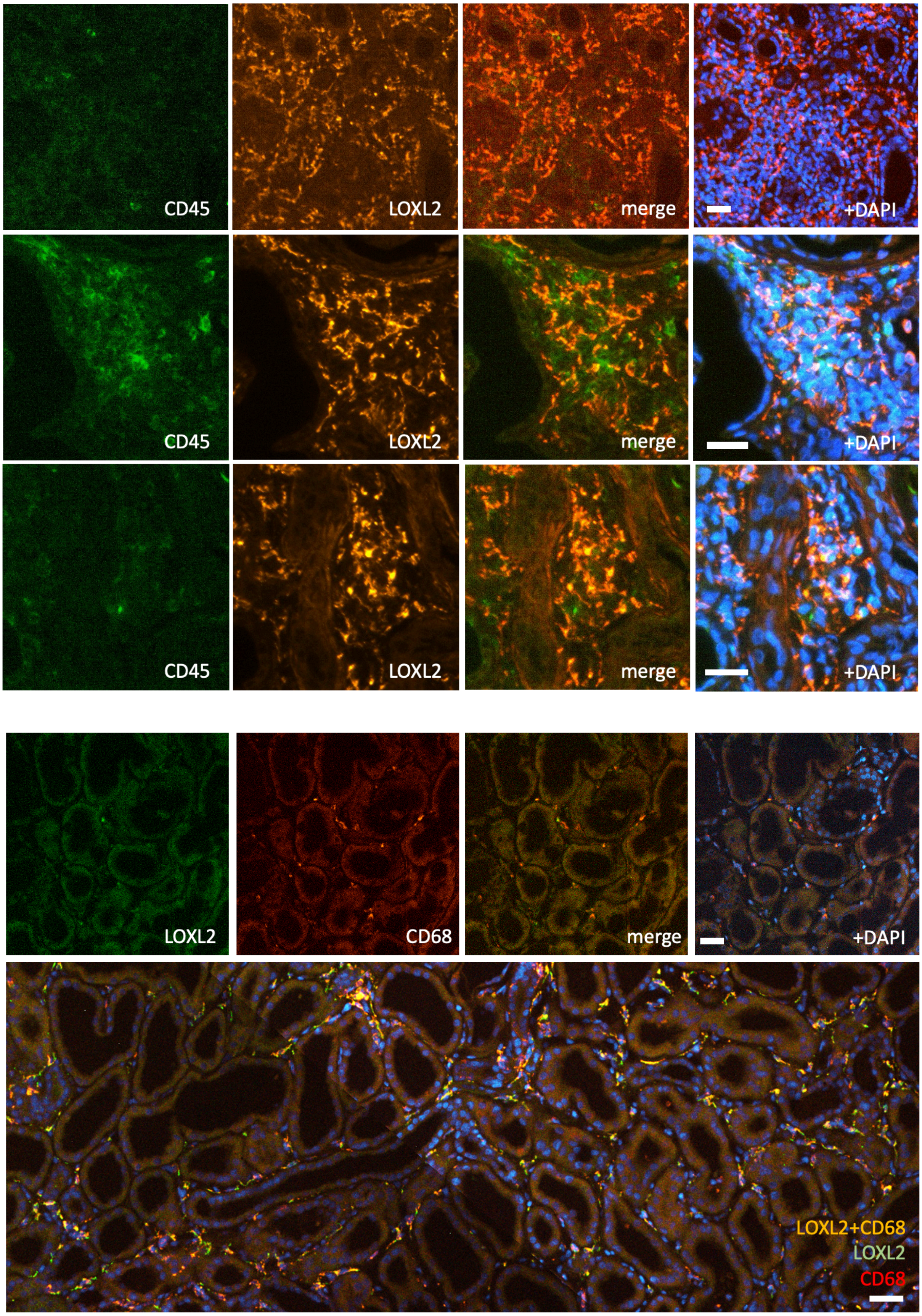
CD45
LOXL2
merge
+DAPI
CD45
LOXL2
merge
+DAPI
CD45
LOXL2
merge
+DAPI
LOXL2
CD68
merge
+DAPI
LOXL2+CD68
LOXL2
CD68

Supplemental data 4: Supplemental information for the single cell analysis shown in main figure 1. a) Patient characteristics of 18 kidney allograft biopsies used for single cell sequencing. b) Quality controls for RNA sequencing using the BD Rhapsody platform. c) Principal component analysis of all analyzed genes in all samples. d) Cell cluster distribution across all biopsies. e) Quantification and variability of mesenchymal, epithelial and leukocyte cell clusters.

a

**samples**	18
**patient characteristics**	
male	16
female	2
age in years	
median (min-max)	50,5 (20 – 78)
days post transplant	
median (min-max)	913 (10 – 9682)
creatinine	
median (min-max)	2,35 (1,26 - 8,82)
donor specific antibodies	
yes	4
no	7
possibly	4
no information	3
Banff diagnosis	
non-rejection	6
ABMR	2
TCMR	8
mixed	2
IFTA	
none	1
low	10
medium	1
high	3
unclear	3

b

**multiplexed samples**	**H4**	**H5**	**H6**
Biopsies (n)	3	8	7
Cells loaded on cartridge	25000	13000	13000
Cell Count (Seq)	6762	3918	4928
Total reads (mRNA)	29 x 10^6	42 x 10^6	35 x 10^6
Total reads (Abseq)	149 x 10^6	171 x 10^6	194 x 10^6
Total reads (Sample Tags)	3 x 10^6	3 x 10^6	5 x 10^6
Median Mols per Cell (mRNA) (DBEC)	390	274	165
Median Mols per Cell (Abseq) (DBEC)	846	495	899
Multiplets (%)	2,6	5,4	5,6
Undetermined (%)	15,3	16,2	9,3

c

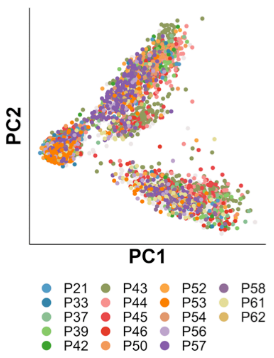

d

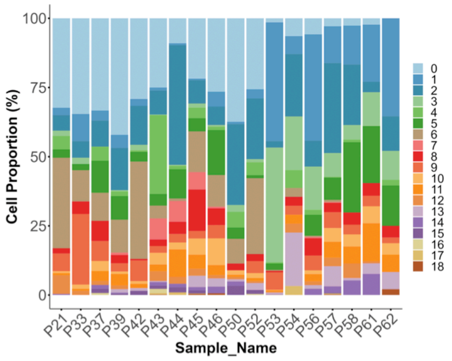

e

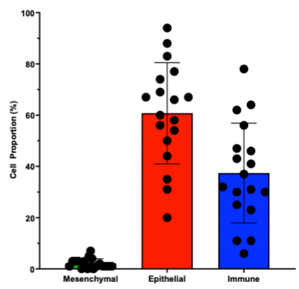

Supplemental data 5: differentially expressed genes in cluster 12. refers to fig. 1

DEG cl. 12	*p_val*	*avg_log2FC*	*p_val_adj*
RGS5	7.835E-197	7.85203291	3.369E-194
EMCN	0	6.93146216	0
ACKR1	6.782E-118	6.27834892	2.916E-115
IFI27	7.597E-274	6.14623787	3.267E-271
COL3A1	3.378E-191	5.63610918	1.453E-188
IL33	1.682E-140	5.62973017	7.231E-138
CD34	1.128E-243	5.59799064	4.851E-241
COL1A2	9.15E-129	5.24125184	3.934E-126
MGP	1.249E-243	5.23250578	5.369E-241
CALD1	7.05E-277	5.13925603	3.031E-274
LRRC32	4.306E-227	4.94351441	1.851E-224
FN1	5.719E-260	4.85817231	2.459E-257
C7	7.4546E-79	4.76996708	3.2055E-76
AQP1	7.632E-103	4.42590328	3.282E-100
SLIT3	5.005E-130	4.39425538	2.152E-127
COL1A1	8.8503E-63	4.14554667	3.8056E-60
COL14A1	3.666E-102	4.04503525	1.577E-99
CFH	2.956E-132	4.01467396	1.271E-129
PDGFRB	4.1628E-93	3.89085431	1.79E-90
CPE	1.808E-128	3.76037044	7.774E-126
NOTCH3	5.269E-136	3.68335237	2.266E-133
CD36	5.7115E-43	3.36919839	2.4559E-40
NRP1	9.8E-108	3.32222046	4.214E-105
HEG1	2.1213E-93	3.24393512	9.1216E-91
DLC1	1.5378E-79	3.1717189	6.6123E-77
ENTPD1	1.1412E-88	3.14238748	4.9073E-86
HIGD1B	1.1177E-74	3.07093352	4.8063E-72
C1S	1.6239E-29	3.0431714	6.9827E-27
IL3RA	1.8561E-51	2.99049195	7.9813E-49
TCF4	3.814E-29	2.64309953	1.64E-26
IFITM3	1.088E-277	2.62962236	4.678E-275
IGFBP5	3.1636E-32	2.43616858	1.3603E-29
LGALS1	1.9052E-96	2.43534425	8.1922E-94
ICAM1	1.4033E-33	2.41283557	6.0341E-31
MYC	3.9395E-30	2.36492165	1.694E-27
CCL2	2.222E-16	2.26755537	9.5545E-14
LOXL2	4.1055E-24	1.95613217	1.7654E-21
IFITM2	1.14E-111	1.95563598	4.902E-109
GIMAP5	5.8855E-18	1.64548625	2.5308E-15
CNTNAP3	5.6096E-10	1.60161721	2.4121E-07
TNFSF10	2.3133E-29	1.58293895	9.9472E-27
LAP3	5.0676E-50	1.52889529	2.1791E-47
SOCS3	2.7714E-13	1.52187113	1.1917E-10
CD40	7.0542E-15	1.51664818	3.0333E-12
IL15RA	1.2424E-08	1.42907907	5.3425E-06
ZBTB16	1.9183E-12	1.40503971	8.2486E-10
GNAI2	5.4179E-26	1.36985091	2.3297E-23
ATP1B3	1.4662E-30	1.35034709	6.3048E-28
LEF1	1.7096E-16	1.24194653	7.3513E-14
CCND2	1.813E-06	1.19247342	0.0007796
TGFBI	2.1764E-11	1.13368284	9.3584E-09
GPX3	1.556E-22	1.12505026	6.6907E-20
LGALS9	7.0757E-05	1.00019992	0.03042542
FOXO1	3.2807E-06	0.97619067	0.00141069
IFNGR1	6.3029E-14	0.96949542	2.7102E-11
CD9	9.033E-23	0.95883701	3.8842E-20
COL6A1	5.0196E-12	0.95160225	2.1584E-09
CD74	4.5889E-26	0.94936634	1.9732E-23
EGR1	9.9645E-08	0.93794834	4.2847E-05
STAT3	1.1668E-11	0.91780031	5.0174E-09
JUN	1.674E-08	0.89113082	7.1981E-06
GIMAP2	1.7856E-06	0.88292222	0.00076779
LGALS3	1.941E-19	0.81459178	8.3463E-17
LAMP1	1.3811E-16	0.72092534	5.9388E-14
STAT5A	6.245E-05	0.71503536	0.0268536
CD63	5.5338E-34	0.65501206	2.3795E-31
YBX3	1.5927E-12	0.62741857	6.8485E-10

ANXA5	7.8504E-20	0.62304662	3.3757E-17
HLA.DMA	8.7412E-08	0.62095269	3.7587E-05
S100A4	3.264E-07	0.58687148	0.00014035
HLA.DPA1	6.0374E-16	0.54179151	2.5961E-13
PDIA6	1.3004E-05	0.46136082	0.0055916
HLA.A	1.4425E-12	0.45134431	6.2029E-10
GAPDH	1.6839E-10	0.43546047	7.2409E-08
KDELR1	7.4092E-05	0.26990766	0.03185946
HLA.DRA	5.1028E-15	0.26421792	2.1942E-12
FYN	8.9538E-05	0.25819548	0.03850135

Supplemental data 6

Gene list for targeted scRNA sequencing:

ADA, ADGRE1, ADGRG3, AIM2, ALAS2, ANXA5, AOC3, APOBEC3G, APOE, AQP9, ARG1, ARL4C, ATF6B, AURKB, AZU1, B3GAT1, BACH2, BAX, BCL11B, BCL2, BCL2A1, BCL6, BIN2, BIRC3, BLK, BLNK, BPI, BTG1, BTLA, C10orf54, C1QA, C1QB, CASP5, CBLB, CCL1, CCL13, CCL17, CCL19, CCL2, CCL20, CCL22, CCL3, CCL4, CCL5, CCND2, CCR1, CCR10, CCR2, CCR3, CCR4, CCR5, CCR7, CCR8, CCR9, CD14, CD160, CD163, CD1A, CD1B, CD1C, CD2, CD2, CD200, CD209, CD22, CD24, CD244, CD247, CD27, CD274, CD28, CD300A, CD33, CD34, CD36, CD37, CD38, CD3D, CD3E, CD3G, CD4, CD40, CD44, CD48, CD5, CD52, CD6, CD63, CD69, CD7, CD70, CD72, CD74, CD79A, CD79B, CD80, CD86, CD8A, CD8B, CD9, CEACAM8, CHI3L1, CHI3L2, CLC, CLEC10A, CLEC4D, CLEC4E, CMKLR1, CMTM2, CNOT2, CNTNAP3, CPA3, CR2, CSF2, CSF3, CST7, CTLA4, CTSD, CTSG, CTSW, CX3CR1, CXCL1, CXCL10, CXCL11, CXCL13, CXCL16, CXCL2, CXCL3, CXCL5, CXCL8, CXCL9, CXCR1, CXCR2, CXCR3, CXCR4, CXCR5, CXCR6, DEFA3, DEFA4, DOCK8, DPP4, DUSP1, DUSP2, DUSP4, EBF1, EGR1, EGR3, ELANE, ENTPD1, EOMES, EPX, F13A1, F5, FAM129C, FAM65B, FAS, FASLG, FCER1A, FCER1G, FCER2, FCGR3A, FCN1, FLT3, FN1, FOSB, FOSL1, FOXO1, FOXP1, FOXP3, FTH1, FUT4, FYB, FYN, GAB2, GAPDH, GIMAP2, GIMAP5, GNAI2, GNLY, GZMA, GZMB, GZMH, GZMK, HAVCR2, HLA-A, HLA-DMA, HLA-DPA1, HLA-DQB1, HLA-DRA, HMMR, ICAM1, ICOS, IER3, IFITM2, IFITM3, IFNA1, IFNG, IFNGR1, IGBP1, IGHA1, IGHD, IGHE, IGHG1, IGHG2, IGHG3, IGHG4, IGHM, IGKC, IGLC3, IKZF1, IKZF2, IL12A, IL12RB1, IL12RB2, IL13, IL15, IL15RA, IL17A, IL17F, IL18, IL18R1, IL18RAP, IL1B, IL1R2, IL1RL1, IL1RN, IL2, IL21, IL22, IL23R, IL25, IL2RA, IL2RB, IL3, IL31, IL32, IL33, IL3RA, IL4, IL4R, IL5, IL6, IL7R, IL9, IRF4, IRF8, ITGA4, ITGAE, ITGAM, ITGAX, ITGB2, JCHAIN, JUN, JUNB, KCNE3, KDELR1, KIAA0101, KIR2DL1, KIT, KLRB1, KLRC1, KLRC3, KLRC4, KLRF1, KLRG1, KLRK1, LAG3, LAIR2, LAMP1, LAMP3, LAP3, LAT, LAT2, LCK, LEF1, LGALS1, LGALS3, LGALS9, LIF, LILRB4, LIPA, LRRC32, LTA, LTB, LY86, LYN, MCM2, MCM4, MGST1, MITF, MKI67, MME, MMP12, MMP9, MS4A1, MYC, MZB1, NAMPT, NCAM1, NCR3, NINJ2, NKG7, NRP1, NT5E, PASK, PAX5, PCNA, PDCD1, PDIA4, PDIA6, PI3, PIK3AP1, PIK3IP1, PMCH, POU2AF1, PRDM1, PRDM1, PRF1, PSEN1, PTGDR2, PTPRC, PTTG2, QPCT, RGS1, RNASE2, RNASE6, RORA, RORC, RPN2, RUNX3, S100A10, S100A12, S100A9, SELL, SELPLG, SLC25A37, SLC7A7, SNCA, SPP1, STAT1, STAT3, STAT4, STAT5A, STAT6, TARP, TBX21, TCF4, TCF7, TCL1A, TGFB1, TGFB3, TGFBI, THBD, THBS1, TIAF1, TIGIT, TLR2, TLR7, TLR8, TLR9, TMEM97, TNF, TNFRSF13C, TNFRSF17, TNFRSF25, TNFRSF4, TNFRSF8, TNFRSF9, TNFSF10, TNFSF13, TNFSF13B, TNFSF14, TNFSF8, TOP2A, TPSAB1, TRAC, TRAT1, TRBC2, TRDC, TREM1, TRIB2, TSPAN32, TXK, TYMS, UBE2C, VEGFA, VMO1, VNN2, VPREB3, VPS28, VSIG4, XBP1, YBX3, ZAP70, ZBED2, ZBTB16, ZNF683, ACKR1, ADH1B, AGXT, ALDOB, AQP1, AQP2, AQP3, ASPN, ATP1B3, C1QC, C1S, C7, CALD1, CCDC80, CCL21, CDH11, CDH6, CEBPA, CFH, CKB, CLU, COL14A1, COL1A1, COL1A1, COL1A2, COL2A1, COL3A1, COL4A1, COL5A1, COL6A1, COL7A1, CPE, CUBN, DAAM1, DCN, DCXR, DEFB1, DLC1, DMRT2, EMCN, ERBB4, ERP27, FABP1, FCGR2B, FGF1, FOXC1, FXYD4, GATA3, GLYAT, GPX3, GRM5, HEG1, HIGD1B, HSD11B2, IFI27, IGFBP5, IRX3, LAMA2, LILRA5, LOX, LOXL1, LOXL2, LOXL3, LOXL4, LRP2, LYZ, MEG3, MFAP5, MGP, MIOX, MOXD1, NOS2, NOTCH3, NPHS2, NUPR2, OGN, PCSK1N, PDGFRA, PDGFRB, POSTN, PTGER1, PTPRQ, RBP4, RGS5, S100A2, S100A4, S100A6, S100A8, SLC4A1, SLIT3, SLPI, SOCS3, SOST, TACSTD2, THBS2, TNC, TYROBP, UMOD, WT1
